# Roles of cancer-associated fibroblasts (CAFs) in anti- PD-1/PD-L1 immunotherapy for solid cancers

**DOI:** 10.1186/s12943-023-01731-z

**Published:** 2023-02-10

**Authors:** Liping Pei, Yang Liu, Lin Liu, Shuochen Gao, Xueyan Gao, Yudi Feng, Zhenqiang Sun, Yan Zhang, Chengzeng Wang

**Affiliations:** 1 Department of Ultrasound, The First Affiliated Hospital of Zhengzhou University, Zhengzhou, 450052 Henan China; 2 Henan Institute of Interconnected Intelligent Health Management, The First Affiliated Hospital of Zhengzhou University, Zhengzhou, 450052 Henan China; 3 Department of Radiotherapy, Affiliated Cancer Hospital of Zhengzhou University, Henan Cancer Hospital, Zhengzhou, 450008 China; 4 Department of Neurology, The First Affiliated Hospital of Zhengzhou University, Zhengzhou, 450052 Henan China; 5 Department of Colorectal Surgery, The First Affiliated Hospital of Zhengzhou University, Zhengzhou, 450052 Henan China

**Keywords:** Cancer-associated fibroblasts, PD-1/PD-L1 inhibitors, Immunotherapy

## Abstract

In recent years, breakthroughs have been made in tumor immunotherapy. However, tumor immunotherapy, particularly anti-PD-1/PD-L1 immune checkpoint inhibitors, is effective in only a small percentage of patients in solid cancer. How to improve the efficiency of cancer immunotherapy is an urgent problem to be solved. As we all know, the state of the tumor microenvironment (TME) is an essential factor affecting the effectiveness of tumor immunotherapy, and the cancer-associated fibroblasts (CAFs) in TME have attracted much attention in recent years. As one of the main components of TME, CAFs interact with cancer cells and immune cells by secreting cytokines and vesicles, participating in ECM remodeling, and finally affecting the immune response process. With the in-depth study of CAFs heterogeneity, new strategies are provided for finding targets of combination immunotherapy and predicting immune efficacy. In this review, we focus on the role of CAFs in the solid cancer immune microenvironment, and then further elaborate on the potential mechanisms and pathways of CAFs influencing anti-PD-1/PD-L1 immunotherapy. In addition, we summarize the potential clinical application value of CAFs-related targets and markers in solid cancers.

## Background

For cancer treatment, the advent of immunotherapy has reinvigorated the field. Immunotherapy mainly includes immune checkpoint inhibitors (ICIs), tumor vaccine therapy, oncolytic virus therapy, and adoptive cell therapy (ACT) [1]. Among them, anti-cancer therapies using ICIs have shown great potential in tumor treatment. Although immunotherapy is more effective and more tolerated than conventional and targeted therapies, many patients demonstrate congenital or acquired resistance [2]. Especially for most advanced solid tumors, inhibitor therapy that blocks the binding of Programmed death receptor 1(PD-1) to Programmed death ligand 1 (PD-L1) has become central [3]. Meanwhile, unfortunately, clinical studies have shown that not all patients are sensitive to this approach, with some patients still showing congenital non-response to PD-1/PD-L1 blockade, and some others developing acquired resistance after treatment [4]. Therefore, in-depth research on drug resistance is necessary to improve the efficacy of PD-1/PD-L1 inhibitors. Current studies have shown that the main factors influencing immunotherapy are: the tumor immune microenvironment, the tumor immunogenicity, the dysfunction of MHCs, irreversible T cell failure, and mutations in tumor genes including the interferon-gamma (IFN-γ) signaling pathway [5–7]. Among them, the tumor microenvironment (TME) status has been shown to play a pivotal role in tumor development and will influence the clinical outcome of treatment [1]. The high heterogeneity and dynamics of TME affect the infiltration of immune cells in tumor tissues, thereby affecting the role of PD-1/PD-L1 inhibitors. For example, the infiltration degree of CD8^+^ T lymphocytes in tumor tissues, the expression status of PD-L1 in tumor cells, the number of CD4^+^ T lymphocytes and the composition ratio of other immune cells in the microenvironment [8–10].

In addition, in recent years, the CAFs in TME have received growing attention. CAFs are a core component of the TME and can not only interact with cancer cells but also affect other components of the TME (such as extracellular matrix and immune infiltration) [11]. As a widespread cell population in the matrix, CAFs also secrete a variety of cytokines and extracellular vesicles, and other substances, and then participate in the remodeling of the extracellular matrix (ECM) [12]. There is a wide range of cellular origins, phenotypic and functional heterogeneity for CAFs [13, 14]. In research analysis of various cancers, subgroup types with different functions have been isolated [15–19]. This also demonstrates the heterogeneity of the CAFs population, suggesting that this phenomenon may be present in a wide range of cancers [19, 20]. Different subsets of CAFs regulate immune cells through different signaling pathways, and reveal different mechanisms of these subsets to exert drug resistance in the process of immunotherapy. What’s more, ncRNAs contained in CAFs-derived exosomes in colon cancer, have enhanced tumor cell proliferation and stem cell properties [21]. CAFs have also been reported to promote tumor progression in studies of solid tumors such as breast, pancreatic, prostate, and colorectal cancer (CRC) [22].

In summary, based on the heterogeneity of CAFs and their interaction with TME, the influence mechanism and related pathways of CAFs on PD-1/PD-L1 immunotherapy are described in solid tumors. In addition, this review also describes the markers associated with CAFs and potential combination therapy targets in solid cancers.

### The heterogeneity and subpopulation types of CAFs

The occurrence and development of cancer are dynamic processes. As cancer progress, cancer cells evolve, resulting in tumor heterogeneity. In addition to genetic differences in cancer cells themselves, different components of TME also lead to changes in the state or phenotype of cancer cells by altering signaling pathways in cancer cells. CAFs are a dynamic part of the TME and play a coordinating role between cancer cells and host matrix responses [23]. With the attention to CAFs and the development of single-cell RNA sequencing (scRNA-seq) technology, researchers have gradually revealed the heterogeneity of CAFs in different cancers. In addition to having basic myofibroblast properties, the activated fibroblast also characteristically expresses alpha-smooth muscle actin (A-SMA/α-SMA), fibroblast activation protein (FAP), fibroblast-specific protein 1 (FSP1), and other factors [15, 24]. Recent observations in genetically engineered mouse models and clinical studies have demonstrated the existence of at least two functionally distinct CAFs, cancer-promoting CAFs (PCAFs) and tumor-suppressing CAFs (RCAFs) [17, 25]. For example, in pancreatic ductal adenocarcinoma (PDAC) [25], researchers have identified the presence of fibroblast-activation protein (FAP)^+^ CAFs in the pro-oncogenic form and fibroblast-smooth muscle actin (α SMA)^+^ CAFs in the anti-oncogenic form. These two subpopulations have completely different roles in the progression and immune landscape of PDAC. One experiment in which primary CAFs were co-cultured with colon cancer cells showed that subpopulations of CAFs with different migratory abilities had different gene expression signatures [21]. The differences in function may originate from the diversity of differentiation pathways. Studies has shown that [13], CAFs are differentiated from different progenitor cells, such as resident fibroblasts, circulating bone marrow mesenchymal cells, epithelial-mesenchymal transition (EMT), and endothelial-mesenchymal transition (ENDMT). The differences in these differentiation pathways might largely determine the heterogeneity of CAFs subsets. Furthermore, in a mouse model of breast cancer, mechanoresponsive (MR) CAFs may exhibit a lower differentiation state relative to immunomodulatory (IM) CAFs. By assessing the epigenomic changes of CAFs in tumors, this study suggests that the level of chromatin opening in different clusters may partly determine heterogeneity [26].

In a single cell analysis of 10 common solid cancers, subpopulations of CAFs with common activation characteristics were displayed. These subpopulations mainly overexpress genes related to collagen activation and matrix metalloproteinases. Three major subgroups were defined as cancer-associated myofibroblasts (CAF muscles), inflammatory CAF (CAF infra), and lipogenic CAF (CAF Ardy Wiranata). In addition, minor subgroups include endothelial-to-mesenchymal transition CAF (CAF ends MT), peripheral nerve-like CAF (CAF PN), and antigen-presenting CAF (CAF ap) [27]. These subsets are mainly related to multiple inflammatory pathways, angiogenesis, endothelium-mesenchymal transition, antigen presentation, etc. This suggests that the potential mechanism of CAFs subsets in promoting tumor progression. However, the subpopulations of CAFs are not always similar in different cancers, and specific subpopulations may exist in certain tumors. For example, in pancreatic cancer [28], researchers have found that a unique Meflin positive subpopulation plays an important role in inhibiting cancer progression.

During the development of cancer, each cluster of CAFs is in dynamic change. The proportion of CAFs subpopulations is not constant. Studies have shown that the number of canonical myofibroblasts (myCAFs, Subset 2) and VEGF^+^ CAFs (vCAFs, Subset 3) cells in breast cancer tissues decreased significantly after transforming growth factor-β(TGF-β) antibody blockade, indicating that these two subsets are very sensitive to this treatment. In addition, the emergence of another new subpopulation, interferon-licensed CAFs (ilCAFs), is refreshing. The cluster is closely related to interferon response signaling pathway and antigen processing and presentation [14]. Similarly, five CAF clusters were identified by scRNA-seq of human skin cancer before and after immunotherapy, of which clusters 3 and 4 (SSL,steady state-like) were present only in pre-treatment CAFs. The proportion of mechanoresponsive (MR) CAFs and immunomodulatory (IM) CAFs increased significantly after treatment. Immunotherapy has been shown to stimulate differentiation of CAFs into other subsets [26]. This also confirmed the homeostasis and plasticity among CAFs subsets.

In addition, the interaction between CAFs subsets and other cells is also different. In spatial transcriptomics analysis of mouse breast cancer models, antigen-presenting CAFs in immune clusters in early tumors colocalized with lymphoid immune cells, while myofibroblast CAFs colocalized with epithelial cells. However, in the later stages of the tumor, immune-associated cell clusters are more closely related to myeloid cells (including macrophage phenotype) and the interactions are more prominent [26]. This also suggests that CAFs subsets are spatially dynamic as tumors progress. Therefore, there are different signal regulation pathways between CAFs and other cells. The ecm-myCAF subset in breast cancer not only increased the proportion of the transcription factor forkhead box protein p3 (Foxp3) ^+^ T cells in the CD4^+^CD25^+^population, but also upregulated the expression of PD-1 and cytotoxic T lymphocyte associate protein-4(CTLA-4) on their surface, which in turn increased the proportion of TGF-β-myCAF [19]. This may reveal the underlying mechanisms of immunosuppression and drug resistance.

### CAFs and tumor immunity

As early as 1909, Paul Ehrlich put forward the original hypothesis of cancer immunity. Cancer immune system distinguishes between “self” and “non-self”, and eliminates “non-self” without damaging “self”, which also forms the original TME concept. Different types of tumors can also promote angiogenesis and stimulate peripheral immune tolerance to form specific microenvironments. With the occurrence and development of cancer, various cellular and molecular mechanisms in the TME are also in the process of dynamic change. Over time, tumor tissue becomes resistant to immunotherapy, which is also the reason why some patients have a poor response to immunotherapy [3]. Among them, immune cells infiltrated in tumor tissue are an important part and an important factor in predicting patient survival [29]. These immune cells include tumor-associated macrophages (TAMs) and neutrophils that promote tumor development, natural killer cells (NKs), and cytotoxic CD8^+^T cells that resist tumor development [30, 31]. In addition to immune cells, CAFs, as an important component of tumor tissue stromal components, also play an extremely important role in cancer progression. It also occupies an important part of tumor tissue. On the one hand, CAFs affect the function of immune cells by secreting various cytokines and products; on the other hand, as a component of tumor stroma, CAFs form a permeability barrier by remodeling the stroma, thereby reducing the effect of drug therapy [11].

At present, increasing studies have opened up new fields in exploring the role of immune resistance in cancer progression [32]. What’s more, the regulation of CAFs to relieve their inhibitory effect on immune cells and overcome their barrier effect, become a new means of tumor therapy.

### The mechanism of CAFs regulating TME to affect PD-1/PD-L1 inhibitor immunotherapy

**Fig. 1:**
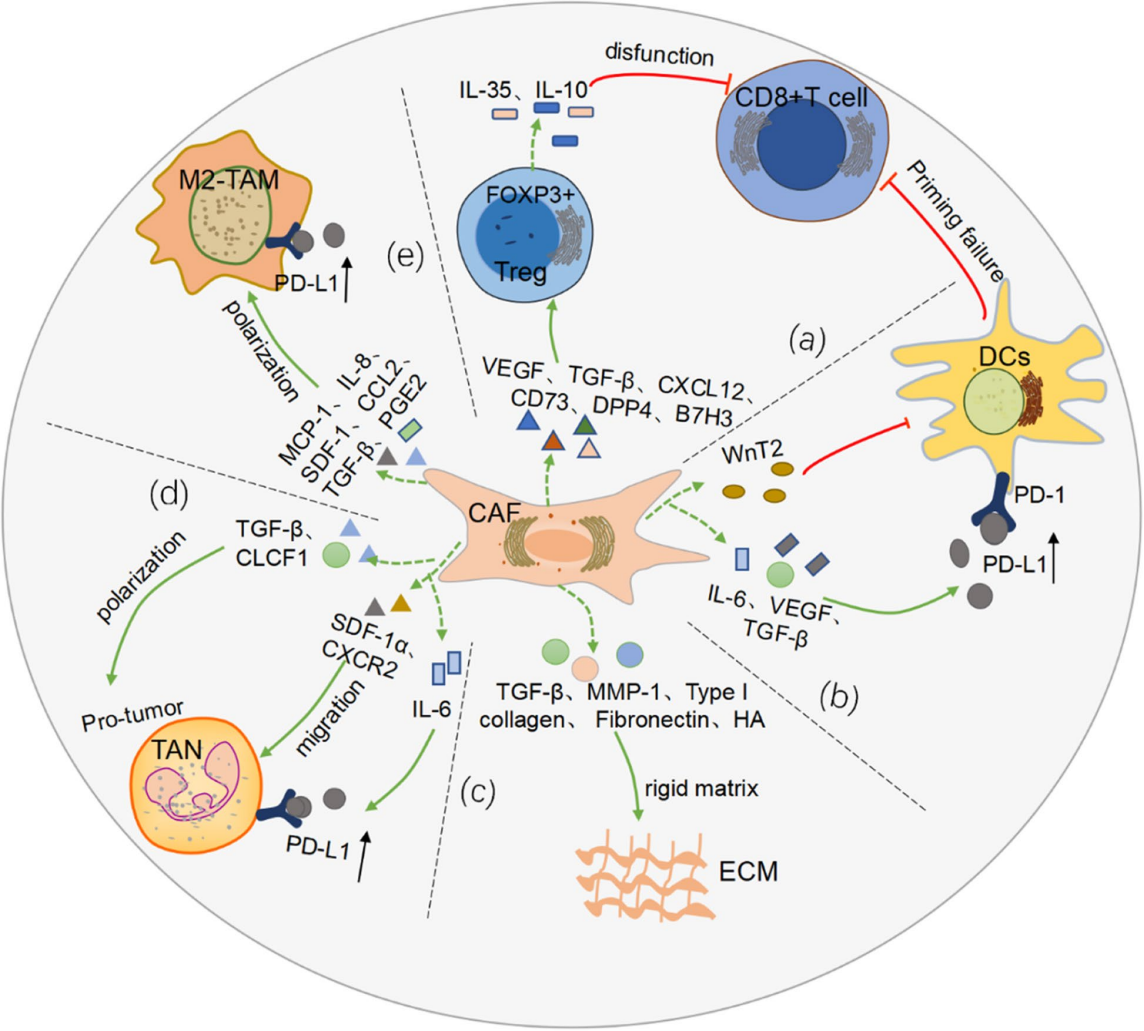
The mechanism of CAFs regulating TME to affect PD-1/PD-L1 inhibitor immunotherapy. **a** CAFs secrete VEGF, TGF-β, CXCL12, etc., which promote Treg recruitment, migration and FOXP3^+^Treg differentiation. The latter promotes CD8^+^ T cell dysfunction by secreting IL-35 and IL-10. **b** CAFs secrete WnT2, etc., thereby inhibiting the anti-tumor response of DC-cell-mediated CD8^+^ T cells. **c** CAFs secrete TGF-β, MMP-1, HA, etc. to remodel ECM, increase rigidity, and prevent immune cell infiltration. **d** TGF-β and CLCF1 differentiate TAN into tumor-promoting types; IL-6 promotes the expression of PD-L1 by TAN, leading to the formation of immune tolerance; SDF-1α and CXCR2 promote TAN migration to tumor tissue. **e** CAFs produce MCP-1, IL-8, SDF-1, etc. to promote monocyte recruitment, induce TAMs to M2 phenotypic differentiation, and impair effector T cell function, and increase the expression level of PD-L1 on the surface of the TAMs, impairing its phagocytosis

As key components of the TME, CAFs are not only involved in the remodeling process of the ECM, but also interact with other cells in the TME [11]. CAFs influence the recruitment and activity of immune cells through direct and indirect regulation of ECM remodeling [33]. For example, CAFs promote immune cell differentiation and enhance immune resistance by secreting a series of cytokines and other effector molecules, including TGF-β, interleukin 6(IL-6), c-x-c chemokine ligand 2(CXCL2), collagen, and laminin, etc. [11]. Moreover, different subsets of CAFs differentially regulate cancer-related pathways and the accumulation of regulatory T cells by interacting with tumor cells [25].Therefore, with an in-depth understanding of the immune microenvironment, people are increasingly aware of the importance of CAFs in affecting immune efficacy. CAFs were summarized the mechanism of regulating the TME and consequently affecting the PD-1/PD-L1 inhibitor immunotherapy (Fig. 1).

#### CAFs-Treg-CD8 + T

Regulatory T cells (Tregs) belong to the immunosuppressive subset of CD4^+^ T cells and are important in maintaining immune homeostasis. Tregs accelerate immune evasion of tumor cells by disrupting tumor immune surveillance and impairing anti-tumor immune responses [34]. The mechanism that a differentiated immunosuppressive Treg phenotype is induced in the TME, remains to be further explored. It is known that in terms of spatial distribution, Tregs are distributed in the tumor stroma in far greater numbers than cancer nests, and CAFs are also usually distributed in the stroma [35]. The spatial intersection leads to mutual crosstalk between the two. On the one hand, CAFs promote Treg recruitment and migration, resulting in increased infiltration at tumor sites. For example, in CRC [36], CD70^+^ CAFs stimulated Treg migration and significantly increased their frequency at tumor sites. In addition, Vascular endothelial growth factor-A (VEGF-A) released by CAFs directly or indirectly participates in the induction and maintenance of Treg cells [37].

On the other hand, CAFs also promote the transformation of Treg cells and are closely related to immunosuppression. In the stromal of lung adenocarcinoma [35], Tregs and CAFs were found to be spatially close, and in vitro experiments showed that CAFs in high-Treg adenocarcinoma had higher secretion capacity of immunoregulatory cytokines, leading to the induction of Treg; and further proved that TGF-β and VEGF were highly expressed in stroma-derived CAFs containing high concentrations of Tregs. CD8^+^ T cells are induced to express Foxp3 in the presence of TGF-β. These FOXp3-positive cells are widely present in the tumor immunosuppressive microenvironment and promote the differentiation of Treg cells [38, 39]. Furthermore, after co-culture of cancer cells and fibroblasts in vitro, the results showed that the expression of COX-2 was induced to increase in CAFs [40]. This enzyme and its product prostaglandin E2 (PGE2) play an important role in Treg function in tumors. PGE2 induces the expression of FOXP3 in Tregs [41]. Additionally, a study on breast cancer [42], indicated that FAP^+^PDGFRβ^+^ CAF in tissues not only released CXCL12 to promote the migration of CD4^+^CD25^+^ T cells, but also expressed CD73, dipeptidyl peptidase IV (DPP4) and B7H3 to promote the conversion of CD4^+^ T cells to FOXP3^+^ Treg cells. Subsequently, Tregs inhibit CD8^+^ T cells function and promote their depletion by producing IL-35 and IL-10 [43, 44]. In summary, CAFs can affect the killing ability of CD8^+^ T cells to tumors by regulating the differentiation of Treg (Fig. 1a).Moreover, Treg cells inhibit the secretion of IFN γ by CD8^+^ T cells, indirectly promoting M2-like TAM dominance, thereby enhancing cancer progression [45].

#### CAFs-DCs-CD8 + T

Dendritic cells (DCs) are the most efficient antigen-presenting cells (APCs), linking innate and adaptive immunity. DCs are phenotypically and functionally heterogeneous under physiological conditions. DC1s (type 1 dendritic cells) are capable of presenting antigens of MHC class I molecules to CD8 T cells, while DC2s (type 2 dendritic cells) are capable of presenting MHC class II molecules to CD4 T cells [46]. CAFs act an essential role in interfering with DC maturation, antigen presentation and immune response. Both TGF-β1 and TGF-β2 are upregulated in CAFs, and the both factors critical for maintaining an activated fibroblast phenotype [47]. TGF-β immobilizes DCs by blocking the migration of DCs, which in turn blocks the transport of antigens to the draining lymphatic system [48]. What’s more, under the stimulation of TGF-β, the expression of MHC-II, CD80, CD40, etc. on the surface of DCs are down-regulated, thereby changing the phenotype, which is beneficial to indirectly participating in the process of immunosuppression [49] (Fig. 1b). Likewise, in hepatocellular carcinoma [50], CAFs recruit normal DCs and induce their differentiation into regulatory DCs(RDCs) and dysfunctional DCs by activating the IL-6-mediated STAT3 pathway. These subpopulations are characterized by low levels of expression of costimulatory molecules and little antigens are present, while suppressive cytokines such as indoleamine 2,3-dioxygenated (IDO) are secreted. In addition, VEGF released by CAFs participates in the abnormal differentiation process of DCs and impairs their antigen-presenting function by inhibiting the activation of nuclear factor kappa-B(NF-κB) [51]. Strikingly, VEGF also promotes immune tolerance by upregulating the expression of PD-L1 on the DC surface [52]. In a related study of primary esophageal squamous cell carcinoma (ESCC), we found that WNT2^+^ CAFs were negatively correlated with CD8^+^ T cells. The further mechanistic analysis confirmed that WNT2 secreted by CAFs, inhibited DCs-mediated antitumor T cell responses through the SOCS3/p-JAK2/p-STAT3 signaling cascade [53] (Fig. 1b).

#### CAFs-ECM

The ECM is a complex network of extracellular proteins, proteoglycans, and glycoproteins [54]. As an important component of TME, ECM plays a significant role in tumor growth, migration and metastasis [55]. Studies showed that in the process of CAFs activation, the extracellular matrix continuously deduced the process of remodeling through matrix deposition and protein degradation [56]. This gives solid tumors rich in CAFs an “innate advantage” to form a rigid matrix. With the in-depth study of ECM remodeling, the researchers found that [57, 58], CAFs played an essential role in matrix remodeling by secreting a variety of matrix proteins (such as type I collagen, fibronectin, matrix metalloproteinase-1(MMP-1)) and releasing cytokines(Fig. 1c). For example, the growth factor TGF-β1 released by CAFs was found to be indispensable in the regulation of matrix remodeling [59]. In another animal experiment [14], a substantial reduction in the overall pro-fibrotic program in tumors of mice treated with anti-TGFβ. Furthermore, it was reconfirmed by analyzing the genetic signatures associated with collagen deposition and fibrosis. More in-depth research revealed that TGF-β signaling activation in CAFs was associated with transcription program dysregulation. Tumors activating the program carry characteristic gene profiles, such as BRAF, TP53 mutations, and MYC amplification, which are effective indicators of PD-1 blockade failure [59]. Hyaluronic acid is also often found to be excessively deposited in malignant tumors. The study reported that fibroblasts deficient in the hyaluronan synthase gene (HAS2) showed severe impairments in recruiting macrophages, and the HA-deficient stromal environment was also detrimental to tumor vascular and lymphangiogenesis [60].

The ECM modified by CAFs also modulates the activity of other immune cell populations. Complex crosstalk between cancerous collagen matrix and TAMs [61]. On the one hand, CAFs are closely related to the collagen-rich matrix, which not only promotes the activity of monocytes, but also induces the polarization process of M2 macrophages [62]. On the other hand, the induced formation of TAMs regulates collagen deposition, thereby increasing matrix rigidity [61]. Some studies showed [63, 64] that accumulation of collagen density or hardness activates the FAK pathway. This would not only drive CD8^+^ T cell depletion, but also promote the recruitment of other immune cells, such as Tregs and MDSCs. This process is closely related to the formation of immunosuppressive TME. It is undeniable that changes in matrix composition affect the migration and spatial differences of immune cells [65]. However, the specific crosstalk mechanism between ECM and immune cells still remains to be further explored.

#### CAFs-TANs

Similar to most immune cells in the TME, tumor-associated neutrophils (TANs) also exhibit diverse phenotypic and functional heterogeneity, influenced by other cells in the TME. According to different polarization states, neutrophils are divided into N1 type with anti-tumor function and N2 type with tumor-promoting effect [66]. CAFs affect TAN polarization by releasing a variety of cytokines. Among them, cardiotrophin-like cytokine 1 (CLCF1) released by CAFs is very important in inducing the polarization of tumor-promoting TANs, which is achieved by up-regulating the expression of CXCL6 and TGF-β in hepatoma cells [67] (Fig. 1d). What’ more, some studies confirmed that [68], in addition to TGF-β induction of a population of TANs that develop a tumor-promoting phenotype, blocking TGF-β results in the recruitment and activation of TANs with an anti-tumor phenotype. In addition to inducing polarization, CAFs also recruit surrounding neutrophils to aggregate into tumors, among which stromal-derived factor-1 alpha(SDF-1α),CXCR2 secreted by CAFs have been shown to promote the migration of TANs [69, 70] (Fig. 1d). CAFs may be involved in many stages of TANs progression to promote the formation of tumor immunosuppressive microenvironment through various mechanisms.

Remarkably, CAFs-derived IL-6 upregulates PD-L1 expression by activating the JAK-STAT3 signaling pathway of TANs, ultimately impairing T cell function and inducing immune tolerance in hepatocellular carcinoma(HCC) [69] (Fig. 1d). Similarly, in gastric cancer [71], tumor cell-derived MSCs (GC-MSCs) induce chemotaxis and activation of neutrophils by activating the IL-6-STAT3-ERK1/2 signaling pathway. Conversely, neutrophils activated by GC-MSCs induce normal MSCs to differentiate into CAFs. The interaction between GC-MSCs and TANs suggests a new mechanism for the remodeling of the cancer ecological environment. More importantly, by revealing the mechanism of action between CAFs and TANs, it provides a new idea for us to explore the mechanism of PD-1/PD-L1 immune resistance, and shows a new target site for the combined application of targeted drugs. However, due to the limited number of research reports, the specific mechanism of the interaction between the two remains to be further explored in the future.

#### CAFs-TAMs

The main sources of TAMs include peripheral blood monocytes and tissue-resident macrophages. The heterogeneity of TAMs is caused by the combined influence of the complexity of the growth environment and the plasticity of the cells themselves. Moreover, macrophage inflammation may promote tumor immune escape [72]. Notably, the high plasticity of TAMs is tightly regulated by specific chemokines and cytokines, which in turn polarize into a pro-inflammatory ‘M1 type’ or an immunosuppressive ‘M2 type’, upon application of differences in stimulated and induced transcription. Next, the M2 type is further divided into M2a, M2b, M2c, and M2d subtypes [73]. Most TAMs express markers of the M2 type, suggesting that infiltrating macrophages are reprogrammed to a ‘prominent’ phenotype by factors in the TME [74]. TAMs are one of the important components of TME and act an essential role in the regulation of tumor immune microenvironment, especially tumor immunosuppression. TAMs may exert both anti-tumor and tumor-promoting activities, while the molecular mechanisms known so far are very limited [75]. The study demonstrated [76], the proportion of macrophages in “fibrotic” matrices was significantly higher than in “inert” matrices, showing a potential interdependence between immunosuppressive TAMs and activating CAFs. In addition, studies showed that [74, 77, 78], CAFs promoted monocyte (macrophage precursor) recruitment and differentiation into cancer-promoting macrophage subsets (M2-type TAMs), thereby impairing effector T cell responses and inducing immunosuppression in the TME(Fig. 1e). In colon cancer, CAFs-induced M2-type TAMs had high expression of PD-1 on the cell surface, and the expression level was negatively correlated with the efficiency of phagocytosis of tumor cells [79] (Fig. 1e). Similarly, One study reported that the presence of TAMs aggravated the degree of immunosuppression in the pancreatic cancer microenvironment, leading to resistance to PD-1/PD-L1 blockade therapy [80]. CAFs might also inhibit certain aspects of TAM activity. In studies on targeted neuroblastoma models, blocking CAF-derived PGE2 reduced tumor proliferation and promoted the proportion of macrophages to differentiate into M2 type, thereby inhibiting tumor growth [81]. Clinical studies have demonstrated a correlation between accumulation of TAMs and adverse clinical outcomes [82]. In addition to the promotion of TAMs by CAFs, conversely, M2 macrophages also accelerate the progression of EMT by secreting soluble factors such as IL-6 and SDF-1, thereby stimulating the activation of CAFs. In other words, macrophages and CAFs have complex interaction mechanisms [83]. These mechanisms contribute to the formation of an immunosuppressive microenvironment and complicate immunotherapy. These facts lead us to explore the detailed mechanism of CAF-TAM in immune checkpoint therapy, which is also the current need for targeted therapy.

### The related regulatory pathways of CAFs affecting PD-1/PD-L1 inhibitor immunotherapy

**Fig. 2:**
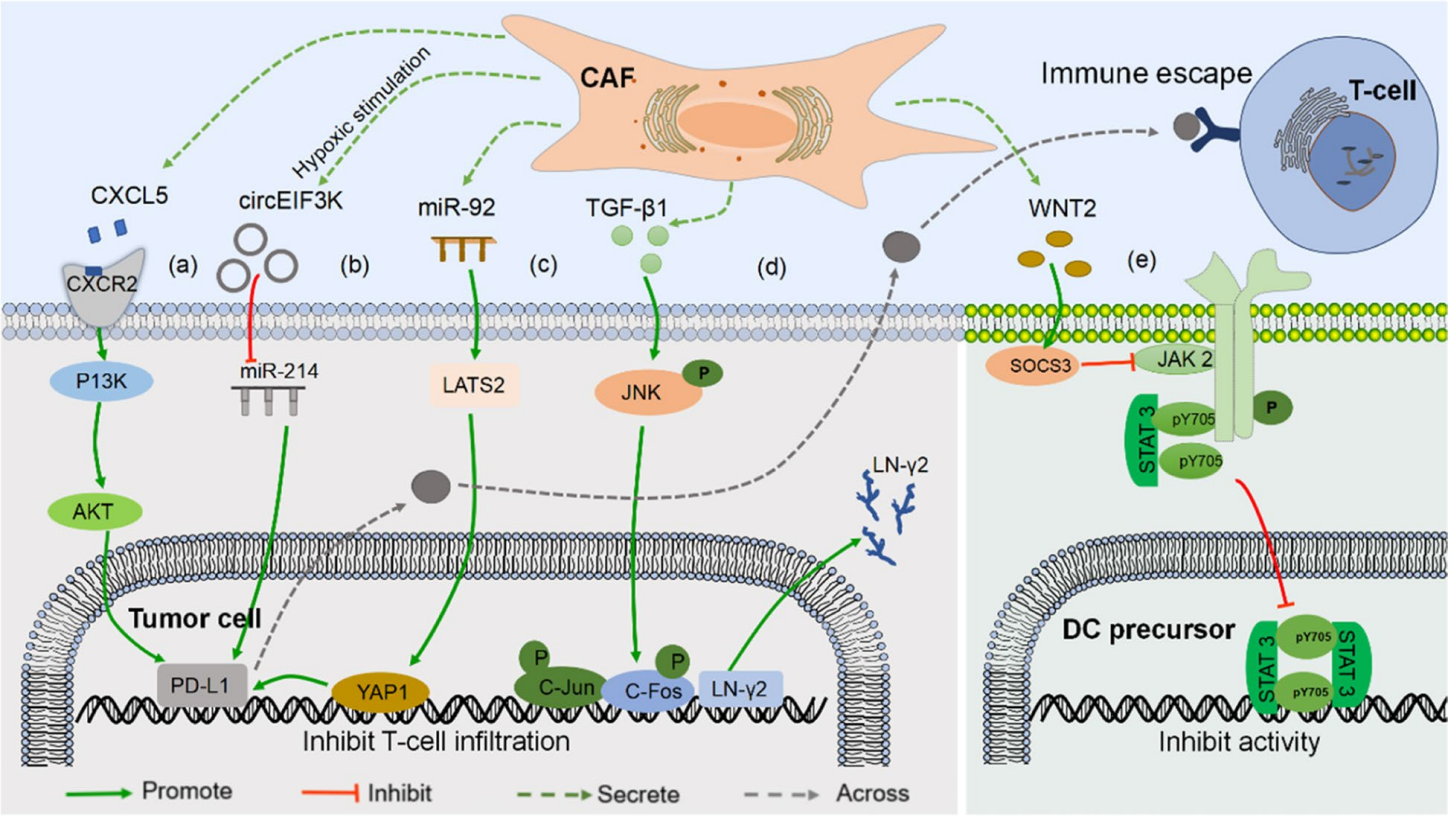
CAFs affect PD-1/PD-L1 inhibitor immunotherapy-related regulatory pathways. **a** WNT2 upregulates SOCS3 on DC precursors, thereby inhibiting the JAK2/STAT3 pathway and blocking DC differentiation and maturation. **b** TGF-β1 induces the expression of LN-γ2 in cancer cells through JNK/AP1 signal transduction, thereby hindering T cell invasion of cancer nests. **c** CAFs secrete CXCL5 to bind to CXCR2 on cancer cells, and then activate the PI3K/AKT pathway to promote PD-L1 expression on the cancer cell surface. **d** After miR-92 is taken up by cancer cells, it acts on LATS2/YAP1 signaling and increases PD-L1 transcriptional activity. **e** Hypoxia induces CAFS secretion of circeIF3K, which acts on the miR-214/PD-L1 axis, ultimately leading to immune evasion

CAFs induce the expression of PD-L1 on cancer cells by deriving some cytokines and vesicles, thereby promoting tumor immune escape. Here, several related regulatory pathways of CAFs affecting PD-1/PD-L1 inhibitor immunotherapy are summarized (Fig. 2).

In mouse melanoma and CRC [84], CAFs secrete CXCL5 to bind to c-x-c motif chemokine receptor 2(CXCR2) on cancer cells. The PI3K/AKT signaling pathway is then activated, promoting PD-L1 expression on the surface of cancer cells. Ultimately, immune evasion occurs (Fig. 2a). In addition, in vitro studies on CRC showed [85], hypoxia induced CAFS to secrete exosomal CirceIF3K, which induced PD-L1 expression in cancer cells, suggesting that this is achieved through the miR-214/PD-L1 axis (Fig. 2b). Moreover, another report is that [86], CAFs-derived exosomal miR-92 induced PD-L1 expression in breast cancer. After miR-92 is taken up by cancer cells, it acts on the target gene large tumor suppressor kinase 2 (LATS2). The latter interacts with transcriptional coactivator Yes-associated protein 1(YAP1) to promote nuclear translocation and binding to enhancers, thereby promoting PD-L1 transcriptional activity (Fig. 2c).

In addition to regulating the expression of PD-L1 on the surface of cancer cells, CAFs also affect immune efficacy by regulating immune cell differentiation and ECM remodeling. For example, in non–small cell lung cancer (NSCLC) and ESCC [16], TGF-β1 transduces the expression of laminin γ2 (LN-γ2) on tumor cells through JNK/AP1 signaling. Then LN-γ2 affects T cell receptor (TCR) gene transcription, which in turn inhibits T cell chemotaxis. This ultimately leads to ineffectiveness of anti-PD-1 therapy. Further analysis revealed that three AP1 (mainly c-jun and c-fos) binding sites were found upstream (− 92 to − 15 nucleotides) on the transcription factor binding site in the promoter region of the LN-γ2 gene. TGF-β1 stimulation significantly increased phosphorylated-c-jun (p–c-jun) and phosphorylated-c-fos (p–c-fos) binding to the LN-γ2 promoter (Fig. 2d). TGF-β1 inhibitors effectively inhibit this process. Therefore, the JNK/AP1 signaling pathway is involved in the upregulation of LN-γ2 mediated by TGF-β1. In addition, CAFs can also be indirectly regulated to weaken the killing effect of T cells. CAFs-derived WNT2 regulates the differentiation of DCs and attenuates the T cell killing effect in tumors. Tyrosine kinases (JAKs) and signal transducers and activators of transcription (STATs) are critical for inducing differentiation. WNT2 inhibits the p-JAK2/p-STAT3 (TYR705) pathway by up-regulating suppressor of cytokine signaling 3 (SOCS3) on DC precursors, thereby blocking DC differentiation and maturation [53] (Fig. 2e).

### Different mechanisms of tumor cells driving CAFs to affect the immune efficacy of PD-1/PD-L1 inhibitors

**Fig. 3:**
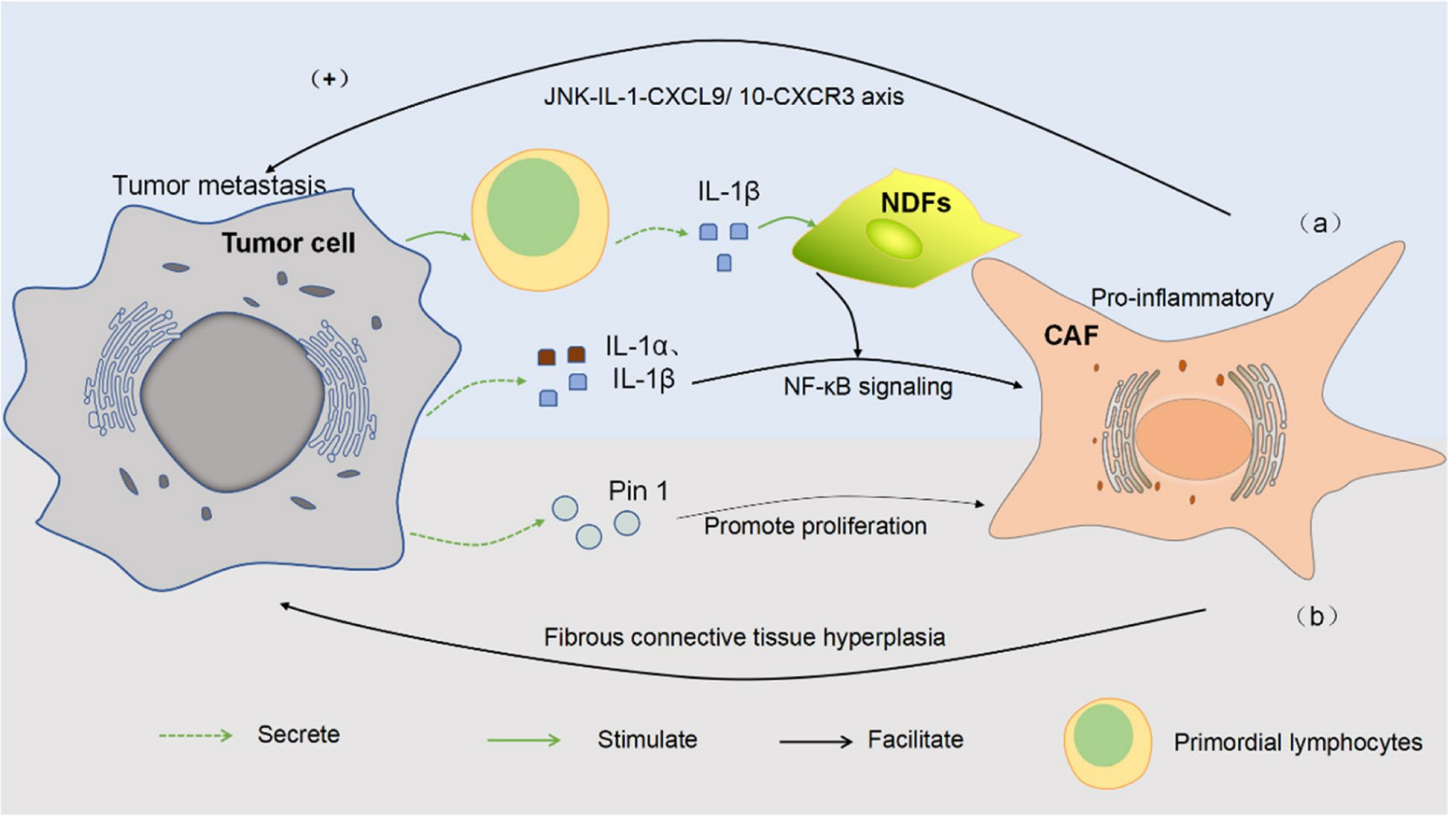
Tumor cells drive CAFs through different mechanisms to affect immune efficacy. **a** By directly secreting cytokines or indirectly stimulating primitive lymphocytes, tumor cells act on the NF-κB signaling pathway, activate fibroblasts, differentiate them into pro-inflammatory phenotypes, and then CAF acts on the JNK-IL-1-CXCL9/ 10-CXCR3 axis to promote tumor metastasis. **b** Pin1 promotes CAF proliferation, promotes fibrous connective tissue proliferation and immunosuppressive TME formation, which in turn is conducive to cancer progression

Tumor cells educate surrounding cells to enhance tumor immunity, thereby supporting their own proliferation, migration, and invasion [87]. In other words, cancer cells exploit the plasticity of stromal cells to enhance a microenvironment conducive to cancer cell growth. This is also one of the reasons for the poor immune efficacy of PD-1/PD-L1 inhibitors. This article summarizes several mechanisms by which tumor cells drive CAFs to affect immune efficacy (Fig. 3).

Related studies have shown that normal skin fibroblasts (NDFs) are “educated” by tumor cells to express pro-inflammatory genes, mainly by activating the NF-κB pathway in activated fibroblasts. This pro-inflammatory process occurs in early tumors. The adaptive immune cells induce the expression of IL-1β by primary immune cells, thus promoting the activation of CAFs [88] (Fig. 3a). Likewise, in CRC [89] and its liver metastases in research [90], NF-κB target genes COX-2 and IL-8 were also found to be highly expressed in human myofibroblasts, suggesting that NF-κB signaling may also play a role in the activation of CAFs in CRC. Another niche study of fibroblasts in metastases found that IL-1α and IL-1β secreted by breast cancer cells induced CXCL9 and CXCL10 production in lung fibroblasts, which was also achieved through NF-κB signaling and ultimately promoted the progression of lung metastasis. Surprisingly, the CXCL9/10-binding chemokine receptor CXCR3 was specifically expressed in a small subset of breast cancer cells, exhibited tumor-initiating capacity when co-transplanted with fibroblasts, and had high JNK signaling Drives IL-1α/β expression. Importantly, disruption of the intercellular JNK-IL-1-CXCL9/10-CXCR3 axis reduced metastatic colonization in xenograft and syngeneic mouse models [91]. In the study of PDAC [92], the researchers analyzed tumor samples and found a unique proline isomerase, Pin1, overexpressed in both CAFs and cancer cells. This study revealed that Pin1 drives both desmoplastic and immunosuppressive TME by acting on CAFs (Fig. 3b). Furthermore, Pin1 binds to the pSer929-Pro site in HIP1R, which in turn promotes actin binding and ultimately drives PD-L1 on cancer cells into lysosomal degradation. In addition, in vivo animal experiments have also shown amazing results. Pin1 inhibitor combined with PD-1 antibody and gemcitabine (GEM) synergistically destroys tumor fibrosis, improves the inhibition of TME, and achieves the level of tumor elimination [92]. Undoubtedly, it is necessary to study the interaction mechanism between tumor cells and CAFs, which provides a new idea for us to study immunotherapy resistance.

### The value of TGF-β in CAFs regulating the immune efficacy of PD-1/PD-L1 inhibitors

The production, storage, and release of TGF-β (transforming growth factor beta) are strictly controlled processes. Active TGF-β stored in the ECM complex is released into the TME through cleavage mediated by various serine proteins or cathepsins, especially the matrix metalloproteinases present in the TME [93]. In contrast, high levels of TGF-β in the stroma are mainly contributed by CAFs [94]. Meanwhile, there is substantial evidence that [95], CAFs contribute to the active release process of TGF-β. However, this point is still not confirmed by in vivo experiments.

In recent years, the study on TGF-β has become growing in-depth, especially its dual roles in the TME. In normal tissues and precancerous cells, TGF-β inhibits cell stagnation, differentiation and apoptosis processes. It also reduces inflammation and interstitial-derived mitocin, thereby maintaining homeostasis and inhibiting tumor progression [96]. Conversely, in tumors, the advantages of TGF-β is utilized by cancer cells, which stimulates fibrosis, promotes EMT and drives tumor metastasis [97]. Dysregulation of the C-ECM (cancer extracellular matrix) transcriptional program is associated with activation of TGF-β signaling in CAFs and with immunosuppression in other immunocompetent tumors [59].

Most tumor cells can secrete TGF-β in the late stage. Once the level of TGF-β increases, it hinders the differentiation of naive T cells to Th1 subsets, promotes their transformation into Treg cells, and inhibits the antigen presentation function of dendritic cells, leading to immune escape of tumor cells [98]. In experimental research [99], they found that TGF-β negatively regulated innate CD4^+^ T cell subsets through the production of the pro-inflammatory cytokine IL-6 and signals to promote colonic epithelial cell survival and proliferation in an inflammatory environment, ultimately enhancing risk of dysplasia. Similarly, in castration-resistant prostate cancer, prostate cancer [100], skin melanoma [101] verified that the inhibition of immune response by TGF-β was achieved by antagonizing Th1 differentiation. In a gene expression profiling study of CRC [102], the predictive power of subpopulations with poor prognosis was not derived from the gene expression of epithelial cancer cells but from stromal cells. Meanwhile, all subtypes with poor prognosis had a gene program induced by TGF-β. In addition, in CRC studies, in genetically engineered mice [94], the results also demonstrated that TGF-β blockade significantly enhanced the effect of antitumor immunity by modulating the CAFs-mediated T cell rejection phenotype. In a mouse model of metastatic urothelial carcinoma (mUC) that replicated this immune rejection phenotype, therapeutic administration of TGF-β blocking antibodies and anti-PD-L1 did reduce TGF-β signaling in stromal cells. That increased the penetration of T cells into the tumor center, ultimately leading to immune escape and strong tumor regression [103].

On the one hand, blocking TGF-β synergistically with anti-PD-L1 reprogrammed peritumoral stromal fibroblasts, increased the abundance of infiltrating T cells in tumors, and in particular significantly enhanced CD8^+^ T effector(TEFF) cell signaling [103]. Therefore, increased immune cell infiltration with TGF-β inhibitors might promote sensitivity to anti-PD-1/PD-L1 immune checkpoint therapy and be particularly useful for treating TGF-β-enriched cancers in the stroma [94]. On the other hand, normalization of aberrant transcriptomes in fibroblasts using blockers of TGF-β signaling, or depletion of CAFs may serve as a potential strategy to enhance checkpoint blockade [59]. Therefore, blocking the TGF-β signaling pathway is conducive to regulate the immune efficacy of PD-1/PD-L1 inhibitors.

### The role of exosomes in CAFs affecting tumor immune efficacy

Exosomes are the main components of extracellular vesicles, with a diameter of 30–150 nm, which contain complex RNA components and proteins [104]. Exosomes are used as delivery vehicles to change the behavior of target cells by loading genes, proteins, drugs and small molecules, etc. [105]. Tumor cell-derived exosomes not only act on immune cells to produce immunosuppressive functions, but also participate in processes such as angiogenesis, matrix remodeling, and tumor cell invasion [106]. Furthermore, a large number of stromal cells (eg, CAFs) in the TME also derives exosomes, which plays an important role in tumorigenesis and development [107]. CAFs-derived exosomes affect the behavior of immune cells and cancer cells by activating different signaling pathways, thereby affecting the effect of immunotherapy. This study summarizes the signaling pathways by which CAFs-derived exosomes affect immunotherapy in several cancers (Table 1).

**Table 1:** CAFs-derived exosomes affect signaling pathways in immunotherapy

Cancer type	Exosomes	Functions	Molecular mechanisms	Reference
Breast cancer	miR-181D-5P	Promote cancer cell proliferation, invasion and accelerate EMT	Target CDX2 and downregulate CDX2 and HOXA5	[108]
	miR-92	Increased apoptosis and impaired proliferation of T cells	miR-92/YAP1-LATS2/PD-L1	[86]
	SNHG3 (lncRNA)	Increase glycolysis metabolism and inhibit immune cell activity	Target miR-330-5p and increase the PKM expression	[109]
Lung cancer	OIP5-AS1	Inhibition of PBMCs-induced killing of lung cancer cells	OIP5-AS1/miR-142-5p/PD-L1 axis	[110]
Colorectal cancer	CircEIF3K	Inhibits colorectal cancer cell proliferation, invasion and tube formation	circEIF3K/miR-214/PD-L1	[85]

In addition to affecting immune efficacy, exosomes also intervene in tumor cell behavior through other pathways, thereby promoting cancer development. Exosomal miR-500a-5p molecule affects the expression level of ubiquitin-specific peptidase 28 (USP28) through targeting transport, and ultimately promote cancer proliferation and metastasis [111]. Studies have shown [112], hypoxia up-regulated CAFs-derived exosomal circHIF1A (circ_0032138) higher than normal CAFs. circHIF1A, acting as a miR-580-5P sponge, enhances stem cell properties of breast cancer cells by regulating miR-580-5p targeting CD44 expression. CAFs containing miR-181d-5p accelerate EMT and promote cancer progression by inhibiting the CDX2/HOXA5 axis [108]. SNHG3 (lncRNA) secreted by CAFs promotes breast cancer cell proliferation by regulating PKM (Pyruvate Kinase M1/M2) at the transcriptional level of cancer cells [109]. In PDAC, CAFs induce tumor cell drug resistance via exosomes [20]. In CRC, CAFs secrete long noncoding RNAs (lncRNAs) LINC00659, which interact directly with miR-342-3P in cancer cells. Though upregulating the expression of annexin A2, it promotes EMT and cancer progression [113]. In addition, miR-92a-3p is also taken up by CRC cells to increase its internal expression level, thereby activating the Wnt/β-catenin pathway and directly inhibiting the downstream targets FBXW7 and MOAP1, ultimately attenuating mitochondrial apoptosis and increasing tumor aggressiveness and chemotherapy resistance [114]. Through in vitro culture and animal experiments, CAFs-derived exosomal opa-interacting protein 5 antisense RNA 1 (OIP5-AS1) inhibited the effect of PBMCs on inducing and killing lung cancer cells through the miR-142-5p/PD-L1 axis, thereby promoting lung cancer progress [110]. Meanwhile exosomes offer potential for cancer diagnosis and treatment.

Some down-regulated miRNAs in CAFs-derived exosomes were also associated with cancer progression. A study on head and neck cancer [115], showed that reduced levels of miR-3188 in CAF-derived exosomes promoted the proliferation of HNC cells and inhibited their apoptosis by inhibiting the expression of B-cell lymphoma 2 in recipient cells in vitro and in vivo. In sequencing analysis of oral squamous cell carcinoma [116], the researchers also found that MiR-34a-5p with low exosome expression was transferred to cancer cells by CAFs. miR-34a-5p binds to its direct downstream target AXL, induces EMT and promotes cancer progression through the AKT/GSK-3β/β-catenin/snail signaling cascade.

In melanoma [117], cancer-derived exosomes carry PD-L1 and can be upregulated by IFN-γ. Upregulated PD-L1 impairs the killing function of CD8^+^ T cells, which in turn mediates immunosuppression. In addition, because exosomes can also be detected in the blood circulation, researchers have long used them as biomarkers in many clinical trial studies to explore the value of exosomes in early cancer diagnosis and prognosis. However, there are still uncertainties about the source of exosomes as biomarkers and exosomes are promising to have broad prospects in cancer liquid biopsy [118].

### The mechanisms of CAFs affecting PD-1/PDL-1 inhibitor immunotherapy in different solid cancers

**Fig. 4:**
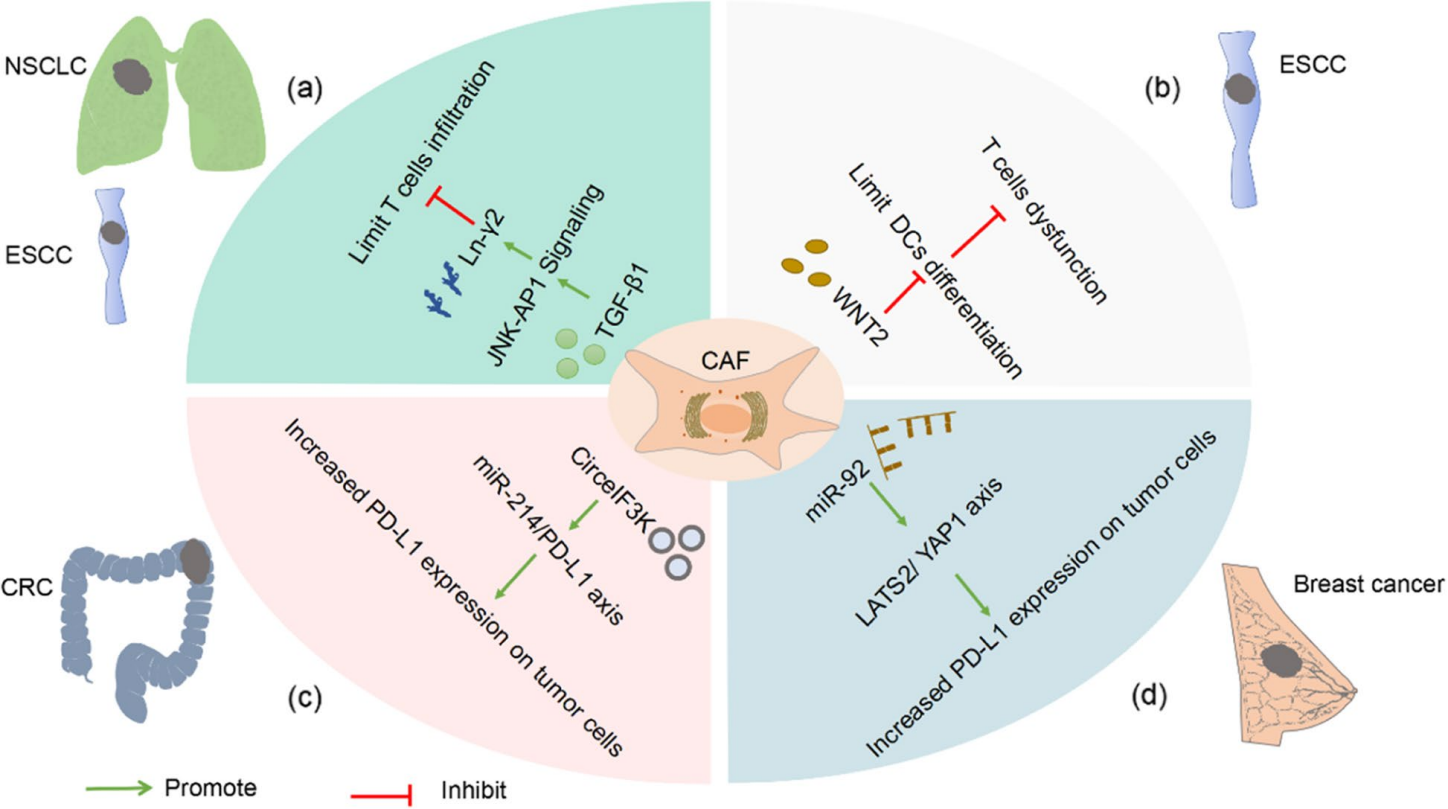
CAFs affect PD-1/PD-L1 inhibitor immunotherapy in different solid cancers. **a** In non–small cell lung cancer (NSCLC) or esophageal squamous cell carcinoma (ESCC), CAFs prevent T cells from penetrating into the cancer nest by promoting the secretion of Ln-γ2 by cancer cells. **b** CAFs-derived WNT2 inhibits the differentiation of DC cells and ultimately impairs the killing of T cells in ESCCs. **c** Hypoxia induces CAFS secretion of CirceIF3K, induces PD-L1 expression in colorectal cancer (CRC) cells through the miR-214/PD-L1 axis, leading to immune escape. **d** Exosome miR-92 acts on the LATS2-YAP1 axis to increase the transcription level of PD-L1 in breast cancer cells. Ultimately, T cell apoptosis and proliferation disorders are induced

Immunotherapy that eliminates tumor cells by inhibiting immune checkpoints to promote anti-tumor immunity has received increasing attention. Antibodies to PD-1 or PD-L1 have proven to change the treatment landscape for many advanced cancers, including melanoma, lung, breast, kidney, etc. A growing number of studies report good long-term results with these treatments compared to traditional treatments. However, in most cases, only 20%-40% of patients respond, and fewer patients achieve long-term disease remission [119]. Different cancer types and stromal heterogeneity are factors that cannot be ignored for the differences in immune efficacy. Since the functions of CAFs in different subgroups are also heterogeneous, the mechanisms of CAFs in different tumor immunotherapy are also different (Fig. 4).

In studies of advanced NSCLC and ESCC [16], they found that TGF-β1 secreted by CAFs utilized the c-Jun N-terminal kinase/activator protein 1(JNK/AP1) signaling pathway to promote the expression of Ln-γ2 in cancer cells, thereby building a protective barrier and limiting the penetration of T cells into the cancer nest (Fig. 4a). In the study of mUC, it was also found that TGF-β could shape the TME by limiting T cell infiltration, thereby suppressing anti-tumor immunity [103]. Besides, heat shock protein 90 inhibitor (XL888) has demonstrated sensitization advantages in immunotherapy for pancreatic cancer. For one thing, it limits PSC/CAF growth and for another, it inhibits JAK/STAT phosphorylation, and reshapes TME in combination with anti-PD-1 therapy [120].

In addition, CAFs also act on immune cells and cancer cells by secreting different cytokines, thereby affecting the immune efficacy. It has been confirmed in animal experiments that WNT2 secreted by CAFs reduces the generation of effector T cells by inhibiting the differentiation of DC cells, and ultimately attenuates the killing effect of T cells in the tumor [53] (Fig. 4b). Additionally, the role of CAFs-derived extracellular vesicles in information transmission should not be underestimated. According to research analysis, CAF-secreted exosomal CirceIF3K inhibits CRC cell proliferation, invasion, and tube formation in vitro, suggesting that the miR-214/PD-L1 axis increases the expression level of PD-L1 in cancer cells [85] (Fig. 4c). In breast cancer [86], exosomal miR-92 acts on the LATS2-YAP1 axis, thereby increasing the transcription level of PD-L1 in cancer cells, ultimately inducing T cell apoptosis and proliferation disorders, and blocking the killing function of NK cells (Fig. 4d).

### The application of CAFs in clinical solid cancer immunity

CAFs are predominantly ‘oncogenic’ in cancer progression. However, in some cases, CAFs also have a tumor suppressor phenotype [17, 25]. Based on the functional heterogeneity of CAFs, prognostic-related markers for different cancers have gradually become the object of attention (Table 2). α-SMA is the most common marker of CAFs and marks activated fibroblasts [121]. Recent studies have reported that it has a dual role in cancer progression. For example, αSMA^+^CAFs promote tumor progression by changing the TME, which is associated with poor prognosis of liver cancer [122]. However, in a study on PDAC, deletion of α-SMA^+^CAFs promotes tumor progression by increasing the number of CD4^+^ FOXP3^+^ Treg cells in tumors [123], suggesting that this subset has an important anticancer role in PDAC. As another well-known biomarker of CAFs, the subset of CAFs with high FAP expression promote cancer cell proliferation, invasion and treatment resistance. Therefore, FAP often indicates poor prognosis of patients [124]. Previous clinical trials have shown that antibodies specific for FAP have shown safe and effective results in phase I trials in advanced cancer [125]. However, in a phase II trial of metastatic colorectal cancer, FAP inhibitors (FAPI) alone did not have a sensitizing advantage over chemotherapy [126]. At present, clinical trials of FAPI are mostly used to improve the diagnostic efficiency of malignant tumors in combination with radioactive tracers and to explore the efficacy in solid cancers in combination with antibodies (Table 3).

**Table 2:** Application of CAFs-related markers in clinical solid cancer immunity

Type of effect	Marker	Immune resistance mechanisms	Clinical application
Double effect	α-SMA	Pro-tumor: promote immunosuppressive microenvironment; Anti-cancer: deletion inhibits immune surveillance and reduces survival	Prognostic indicator
Tumor-promoting	FAP	Promote cancer cell proliferation, invasion, treatment resistance	Preclinical experiments have mixed results, prognostic indicator
	TGF-β	Limit T cell infiltration and suppress antitumor immunity	TGF-β blockade sensitizes anti-PD-L1 immunotherapy and LRRC15 may serve as a prognostic marker
	FGF, PDGF, VEGF	Recruit immunosuppressive cells, promote immune evasion	Nintedanib (BIBF1120) has been used in the clinical trial phase in lung adenocarcinoma
	WNT 2	Disrupting cancer immune surveillance, tumor immune evasion and immunotherapy resistance	Antibodies, inhibitors, activators have been developed and are in clinical trials
Tumor**-**suppressing	Meflin	Negatively correlated with tumor progression	Prognostic potential markers
	VCAN	Inhibits fibroblast proliferation and promotes tumor growth	Potential markers

**Table 3:** Application of CAFs-related markers in clinical solid cancer immunity (Data source: ClinicalTrials.gov: https://beta.clinicaltrials.gov/ provided by the U.S. National Library of Medicine.)

Target	Cancer type	Application phase	Purpose of application	Clinical Trial No
FAP	Breast cancer	Not Applicable	68 Ga conjugated FAPI for targeted FAP and tumor-matrix imaging	NCT05574907
			Diagnostic value of AL18F-fluorodeoxyglucose(18F-FDG)- NOTA-FAPI PET/CT in cancer	NCT05574920
	Gastrointestinal cancers	Phase II	Assess efficacy of 18F-FAPI-74 to detect FAP expressing cells in patients	NCT05641896
	Malignant neoplasms	Not Applicable	The diagnostic value of 68 Ga-FAPI PET/CT in malignant tumors, especially those with low FDG uptake	NCT05034146
			The diagnostic value of 18F-FAPI PET / CT in various cancers and to compare it with 18F-FDG PET / CT	NCT05485792
	Advanced solid tumors	Phase I	Dose escalation study of OMTX7 as a single agent in combination with Pembrolizumab (PD-1 mAb) in patients with advanced solid tumors to assess safety and tolerability	NCT05547321
Meflin	Pancreatic cancer	Phase I/II	To evaluate the safety and tolerability of Am80 in combination with gemcitabine (GEM) and nattaxol in patients with unresectable pancreatic cancer and to determine the recommended dose	NCT05064618
FGFR, PDGFR, VEGFR	Non-small cell lung cancer	Phase I/II	Efficacy and tolerability of Nintedanib in combination with PD-1 antibody and CTLA-4 antibody	NCT03377023
	Advanced solid tumors	Phase Ib	Anti-angiogenic combined with anti-PD-1 therapy to explore the efficacy	NCT02856425
MCT-4, caveolin-1	Breast cancer	Phase I(completed)	To assess the feasibility of the effect of n-acetylcysteine on tumor cell metabolism	NCT01878695
/	Pancreatic cancer	Not Applicable	Establishment of mixed organoid model to predict drug response	NCT05571956, NCT05196334

In addition to traditional biomarkers, other CAFs markers in different cancer types are also being gradually discovered. In a study of primary colon cancer, genes such as IGBP3, OAS2, MX1, and Robo2 were found as prognostic markers in an independent series of colon cancer patients. Meanwhile, studies have shown that the mRNA expression levels of some gene sets with a “CAF signature” appear to be useful for investigating additive effects on patient survival [21]. A subset of TGFβ-driven cells is the most prevalent CAFs in advanced PDAC, and this subset highly expresses a leucine-rich repeat containing 15 protein(LRRC15). While the LRRC15^+^ CAFs signature is associated with adverse responses to immune checkpoint blockade in several different human tumor types [20]. In contrast, studies have shown that Meflin is a marker of a subset of antitumor CAFs in PDAC. Its overexpression in CAFS is inversely correlated with tumor progression. Therefore, the infiltration of Meflin-positive subpopulations often suggests a favorable prognosis in patients [17]. Currently, Am80(common name:Tamibarotene) converts a Meflin negative subpopulation to positive, thereby increasing sensitivity to treatment (clinicaltrials.gov NCT05064618). In addition, versican (VCAN) is also a potential marker of anti-tumor CAFs. VCAN depletion reduces collagen stiffness by inhibiting collagen synthesis and fibroblast proliferation, which in turn promotes tumor growth [127]. These anti-cancer CAFs are beneficial to the remodeling of TME and search for new therapeutic targets.

Some cytokines secreted by CAFs, such as CXCL5, TGF-β, hepatocyte growth factor(HGF), and pro-angiogenic factors, play an important role in promoting malignant transformation and the proliferation and invasion of cancer cells [128–131].

Small molecule inhibitors of tyrosine kinases target fibroblast growth factor receptor (FGFR), platelet-derived growth factor receptor (PDGFR), and VEGF receptor (VEGFR) [132]. Meanwhile, small-molecule tyrosine kinase inhibitors in combination with immune checkpoint inhibitors have been used in phase I/II clinical trials in non-small cell lung cancer (NCT03377023) and phase I studies in advanced solid tumors (NCT02856425). Accumulating findings suggest that aberrant WNT signaling favors disruption of cancer immune surveillance, leading to tumor immune evasion and resistance to immune checkpoint inhibitors [133]. Molecular modulators targeting the WNT pathway have shown promising clinical application potential. It is gratifying that the combined effect of anti-WNT 2 and anti-PD-1 significantly enhanced the anti-tumor response of intratumoral T cells and enhanced the efficacy of anti-PD-1 [53]. This promises us to solve the problem of PD-1/PD-L1 immune resistance. Furthermore, in animal experiments with melanoma and CRC, the up-regulated expression of tumor PD-L1 induced by CAFs was reversed after silencing CXCL5’s receptor CXCR2.This suggests that the CXCL5-CXCR2 axis may be a promising therapeutic target [84].

Based on the above studies, there are differences in the subtypes and functions of CAFs in different cancer types, suggesting that the effectiveness of non-targeted therapy may remain to be tested [12]. It is important to note that biomarkers of opposite function that suggest prognostic cancer progression might be less targeted by immunotherapy.

## Conclusion

In TME, stromal cells crosstalk with immune cells and ECM to promote the formation and stability of an immunosuppressive microenvironment. As an important part of the TME, CAFs stimulate the differentiation and function of immune cells, and ECM in the microenvironment by secreting a variety of cytokines and conducting different signaling pathways. Based on the immunosuppressive effects and related mechanisms of CAFs in resisting PD-1/PD-L1 immunotherapy, researchers have discovered several key molecules, such as TGF-β, Ln-γ2, Wnt2, and exosome molecules. At present, therapeutic strategies of targeting CAFs biomarkers, “star” molecules in the mechanism and limiting ECM remodeling have been developed in clinical experiments, thereby enhancing tumor immune efficacy. Strikingly, the combined targeted blockade approach has seen promising results, namely improved patient survival and enhanced immune efficacy against PD-1/PD-L1. However, the adaptive resistance of solid cancers remains an unsolved problem. The existence of subpopulations and functional heterogeneity of CAFs poses a hurdle for us to elucidate the mechanism. Meanwhile, it also implies that there may be more complex and surprising mechanisms in different tumors. Therefore, in order to improve the precision of combined targeted therapy, finding more reliable and specific anti-tumor CAFs markers in different solid cancers, the rich of fibrous stroma in cancers also has important reference value for the prognosis of solid tumors. In the near future, the deeper mechanisms to promote or activate anti-tumor immune responses remain to be explored in solid cancers.

## Data Availability

Not applicable.

## Abbreviations

PD-1: Programmed death receptor 1
PD-L1: Programmed death ligand 1
IFNγ: Interferon-gamma
TME: The tumor microenvironment
CAFs: Cancer-associated fibroblasts
ICIs: Immune checkpoint inhibitors (ICIs)
ACT: Adoptive cell therapy
ECM: Extracellular matrix
CRC: Colorectal cancer
scRNA-seq: Single-cell RNA sequencing
A-SMA: Alpha-smooth muscle actin
FAP: Fibroblast activation protein
FSP1: Fibroblast-specific protein 1
PDAC: Pancreatic ductal adenocarcinoma
EMT: Epithelial-mesenchymal transition
ENDMT: Endothelial-mesenchymal transition
VEGF: Vascular endothelial growth factor
TGF-β: Transforming growth factor-β
Foxp3: Forkhead box protein p3
CTLA-4: Cytotoxic T lymphocyte associate protein-4
TAMs: Tumor-associated macrophage
NKs: Natural killer cells
IL-6: Interleukin 6
CXCL2: C-x-c chemokine ligand 2
Tregs: Regulatory T cells
PGE2: Product prostaglandin E2
DPP4: Dipeptidyl peptidase IV
DCs: Dendritic cells
APCs: Antigen presenting cells
IDO: Indoleamine 2,3-dioxygenated
NF-κB: Nuclear factor kappa-B
MMP-1: Matrix metalloproteinase-1
HAS2: Hyaluronan synthase gene
TANs: Tumor-associated neutrophil
CLCF1: Cardiotrophin-like cytokine 1
SDF-1α: Stromal-derived factor-1 alpha
HCC: Hepatocellular carcinoma
CXCR2: C-x-c motif chemokine receptor 2
LATS2: Large tumor suppressor kinase 2
YAP1: Yes-associated protein 1
SOCS3: Suppressor of cytokine signaling 3
NSCLC: Non–small cell lung cancer
ESCC: Esophageal squamous cell carcinoma
LN-γ2: Laminin γ2
p–c-jun: Phosphorylated-c-jun
NDFs: Normal skin fibroblasts
GEM: Gemcitabine
TEFF: T effector
mUC: Metastatic urothelial carcinoma
USP28: Ubiquitin-specific peptidase 28
lncRNAs: Long noncoding RNAs
OIP5-AS1: Opa-interacting protein 5 antisense RNA 1
JNK/AP1: C-Jun N-terminal kinase/activator protein 1
LRRC15: Leucine-rich repeat containing 15 protein
VCAN: Versican
